# Multiscale computational modeling of cancer growth using features derived from microCT images

**DOI:** 10.1038/s41598-021-97966-1

**Published:** 2021-09-17

**Authors:** M. Hossein Zangooei, Ryan Margolis, Kenneth Hoyt

**Affiliations:** Department of Bioengineering, University of Texas at Dallas, BSB 13.929, 800 W Campbell Rd, Richardson, TX 75080 USA

**Keywords:** Cancer imaging, Computational biophysics

## Abstract

Advances in medical imaging technologies now allow noninvasive image acquisition from individual patients at high spatiotemporal resolutions. A relatively new effort of predictive oncology is to develop a paradigm for forecasting the future status of an individual tumor given initial conditions and an appropriate mathematical model. The objective of this study was to introduce a comprehensive multiscale computational method to predict cancer and microvascular network growth patterns. A rectangular lattice-based model was designed so different evolutionary scenarios could be simulated and for predicting the impact of diffusible factors on tumor morphology and size. Further, the model allows prediction-based simulation of cell and microvascular behavior. Like a single cell, each agent is fully realized within the model and interactions are governed in part by machine learning methods. This multiscale computational model was developed and incorporated input information from in vivo microscale computed tomography (microCT) images acquired from breast cancer-bearing mice. It was found that as the difference between expansion of the cancer cell population and microvascular network increases, cells undergo proliferation and migration with a greater probability compared to other phenotypes. Overall, multiscale computational model agreed with both theoretical expectations and experimental findings (microCT images) not used during model training.

## Introduction

Cancer is one of the leading causes of death. In 2021 alone, as many as 1.8 million new cases are expected in the United States^1^. This disease imposes a tremendous burden on patients and the healthcare system. It is well established that tumor growth occurs in two distinct phases, which are referred to as the early avascular and late vascular phases. The transition of tumor growth from the relatively harmless avascular phase to the invasive and malignant vascular phase depends upon a key mechanism in cancer development called angiogenesis^2^. Angiogenesis is the process whereby new blood vessels are generated from the existing vasculature to support the metabolic demands of a growing tumor. It is well known that tumors cannot grow beyond a few millimeters in size without angiogenic progression to sufficiently provide oxygen and nutrients to the tumor and disposal of waste products^3^. The delicate interplay between proangiogenic and antiangiogenic signaling molecules controls and maintains tumor angiogenesis. These newly formed blood vessels grow towards the tumor mass and tend to have an abnormal phenotype including gaps between endothelial cells and a tortuous organization^4^. The intrinsic leakiness of the new vessels can cause increased interstitial fluid pressures that interfere with systemic delivery of anticancer drugs and therapeutics. Additionally, the occurrence of impaired blood flow can lead to hypoxia and additional upregulation of proangiogenic biomarkers like vascular endothelial growth factor (VEGF) to further support tumor growth^5^.

An ongoing effort of predictive oncology is to develop a systematic method for predicting the future status of an individual tumor given an optimal representation of the initial conditions and an appropriate mathematical model^6^. To that end, mathematical models of cancer growth have evolved considerably over the last several decades. From cell-based to multi-scale models, these numerical methods can provide valuable insight into the mechanisms underlying tumor growth and the requisite angiogenic process. To help simulate complicated spatiotemporal and multiscale biological behavior as in the case of cancer growth, hybrid modeling approaches have emerged. These hybrid models exploit a combination of continuous and discrete models for each scale^7^, while considering three key phases of cancer development, namely, avascular growth, tumor-associated angiogenesis, and vascular growth. The latter is a hallmark feature of cancer progression^8^. Some current mathematical models of tumor growth are limited in their clinical applicability because they require input data that is nearly impossible to obtain with sufficient spatial resolution in human patients^9^. An emerging strategy to overcome this limitation involves extracting tumor features from medical images and then using them as input parameters to predictive computational models. After model initialization, repeat image-based measurements can be obtained noninvasively and used to update or refine model predictions of cancer growth.

Given the prominent role angiogenesis plays in sustaining tumor growth and systemic drug delivery, it is a logical input parameter to a properly constructed computational model. There are now several clinical imaging modalities that can provide patient-specific data of tumor angiogenesis and at the microvascular scale. Briefly, dynamic contrast-enhanced ultrasound (DCE-US) uses an intravenous contrast agent and real-time US imaging to provide surrogate measures of tumor perfusion^10^ in addition to microvascular volume and morphology^11^. A more recent advance in DCE-US is the introduction of super-resolution US (SR-US) imaging, which can resolve microvascular structures with diameters of tens of micrometers^12^. This new SR-US imaging technique is now advancing to clinical applications beyond preclinical measurements and proof-of-principle studies^13^. Dynamic contrast-enhanced magnetic resonance imaging (DCE-MRI) relies on fast MRI sequences before and after intravenous administration of a contrast agent. Functional analysis of microcirculation depicted in DCE-MRI images provides insight on tumor perfusion and properties of microvascular permeability (i.e., leakage)^14^. DCE-MRI has been used as a biomarker for monitoring responses to chemotherapy and antiangiogenic therapy^15^. Based on blood flow kinetics similar to that used for DCE-MRI, contrast-enhanced computed tomography (CT) imaging can also provide surrogate measures of tumor perfusion^16^. Termed perfusion CT, it was shown in human that tumor blood flow assessed using this noninvasive imaging modality reflects tumor angiogenesis (i.e., microvessel density) and correlated with patient prognosis^17^. If using ultra high-resolution microscale CT (microCT) imaging and an intravascular contrast agent, blood vessels as small as a few micrometers can be resolved in small animal models of cancer^18^. Collectively, these and other technologies represent an assortment of noninvasive methods for longitudinally imaging microvascular networks in cancerous tissue.

To reflect on the direction that the computational modeling field has taken, we proposed extending the definition of multiscale models to include reinforcement learning and an agent-based approach. Using microCT and a preclinical model of breast cancer, we then present a series of computational simulation results that predict solid tumor growth based in part on image-derived model inputs. This image-based computational model made it possible to recapitulate tumor angiogenesis in terms of tumor growth pattern and microvascular network morphology, estimation of diffusible factor gradients (e.g., oxygen and VEGF), and temporal evolution of cell phenotype. Model predictions were compared to repeat images from the same subject. Overall, multiscale computational modeling results were in agreement with both theoretical expectations and experimental microCT image-derived features.

## Results

A multiscale computational model was developed and uses input data from tumor microvascular networks segmented from contrast-enhanced microCT image to simulate several phenomena associated with breast cancer growth. To simplify computational complexity and initial model validation tests, the simulation domain was limited to 2-dimensional (2-D) space. In this model, cells and vessels are represented as individual agents that have their own action (i.e., phenotype). To study the interplay between tumor and microvascular growth, vessels are divided into tip and stalk components. The tumor environment was considered as a rectangular lattice with each element (termed the tumor microenvironment, TME) containing a collection of cells and vessels that receive diffusible factors as input. Moreover, concentration of these factors was calculated by a known diffusion equation to model the interactions between cells and vessels. Throughout the model training phase, a deep reinforcement learning (DRL) technique enabled an agent to learn within an environment and this knowledge was then used to build a dataset for future use. During the testing phase of the simulation, all agents utilized this dataset and the multiscale computational model predicted a preferred action using a neural network approach. Breast cancer-bearing mice were administrated an intravascular contrast agent and microCT images of the tumor and surrounding tissue were acquired at two different time points separated by 1 to 2 wk. After tumor and microvascular segmentation, image data from the first and subsequent microCT scans was used as simulation input for the training and testing phases of the newly developed multiscale computational model.

A prediction-based simulation was implemented in IntelliJ IDEA and written in Java (JetBrains, Prague, Czechia). The geometry of the environment (including cells, vessels and diffusible factors) was a lattice initialized with one million cancer cells (CCs) placed at the domain center with a random phenotype at initialization. All model parameter assumptions were based on theoretical or experimental measures^19,20^. We consider a 2 × 2 cm model region as containing 10^4^ TMEs of size 200 × 200 µm. The simulation time step (termed episode) was set to 1 h, and its duration was fixed at 720 h for both training and test phases. To increase confidence and reduce any model uncertainty effects, the training phase was repeated 50 times by passing the constructed dataset in each repetition to the next repetition. For all 50 repetitions in the training phase, the intratumoral microvascular network was derived from a microCT image from the first mouse acquired at the initial time point. At the 336th episode representing the second time point, repeat microCT images from this same subject were acquired and derived features were also used as model input. During the test phase, microCT image-derived features from a second mouse and at matched time points were utilized. Tumor growth predictions were accomplished using a neural network given the constructed training phase dataset.

The proposed 2-D computational model appeared to be an appropriate approach for simulating cell behavior in the TME and for predicting tumor and microvascular growth. During the test phase and throughout the early stages of modeling, CCs spread from the center of the TME. As oxygen and glucose concentrations at this central location of the tumor decreased, a subset of CCs began to predict a necrotic and hypoxic phenotype, causing some CCs to undergo apoptotic death. However, most CCs were more resistant to oxygen and glucose (i.e., nutrient) deficiencies during simulation, which led to proliferation and an increase in tumor size. Consequently, nutrient concentration in the tumor decreased and aged cells more often reduced selection of a proliferation phenotype. Our prediction-based simulation outcome further showed that a decrease in oxygen availability stimulates CCs to increase VEGF production, consistent with widespread knowledge and the modeling rules adopted.

**Figure 1:**
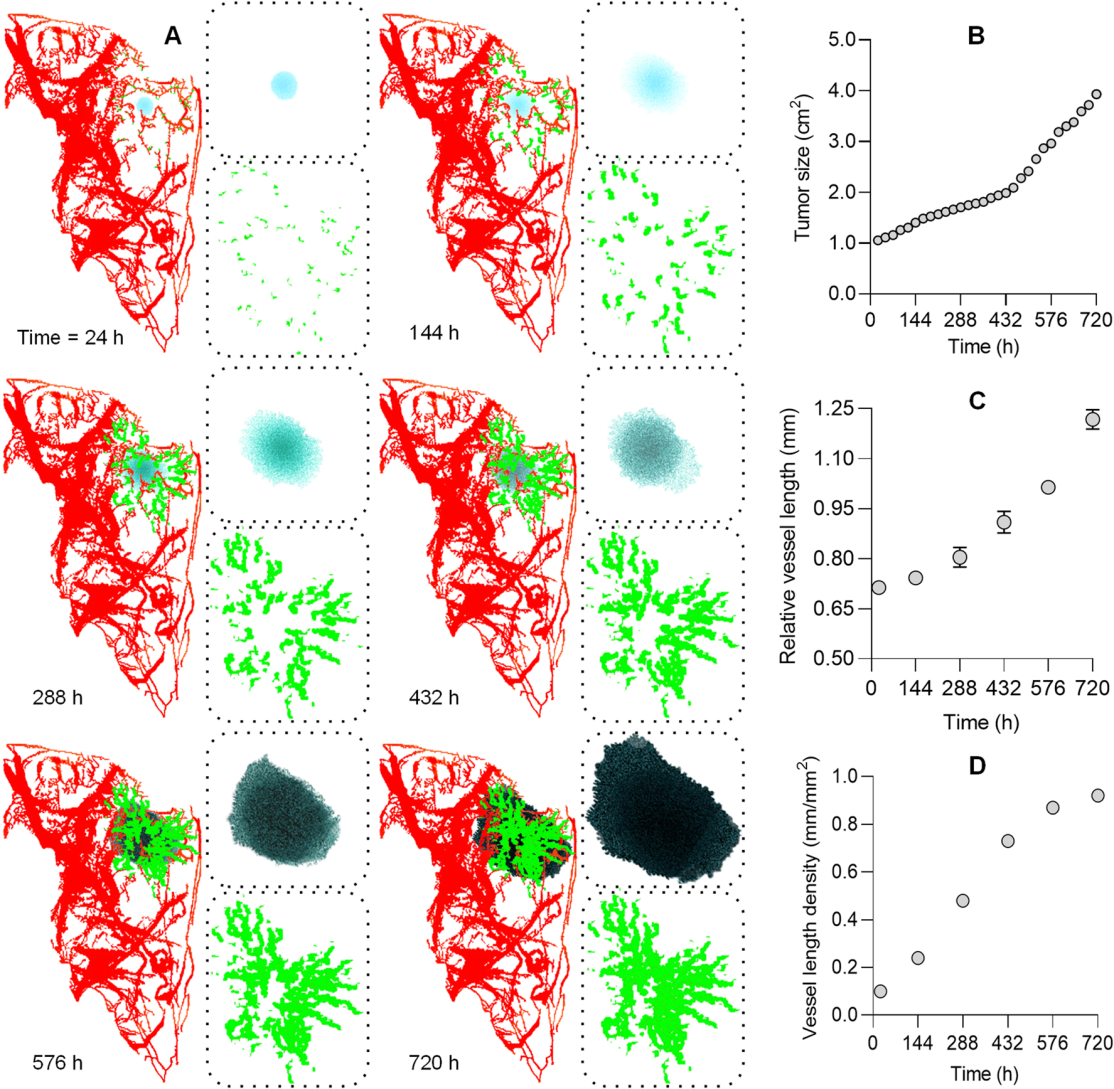
(**A**) Representative multiscale computational model results of tumor growth and microvessel development for a simulation period of 30 d. Model initialization was performed using spatial input data from the in vivo contrast-enhanced microscale computed tomography (microCT) image of the segmented tumor microvascular network (red). New simulated microvascular segments (green) tend to grow toward the tumor location in response to a vascular endothelial growth factor (VEGF) gradient produced by hypoxic cancer cells. (**B**) Simulated changes in tumor size follow the classic exponential growth model. Initially, (**C**) existing vessel length is insufficient to supply the necessary nutrients to hypovascular areas before new microvascular growth, (**D**) leading to increased intratumoral vessel density, which is an indicator of tumor angiogenesis.

During the simulation test phase of microvascular growth, new sprouting microvessels typically grew towards hypoxic CCs, Fig. 1A. Since hypoxia was lowest in the tumor core, sprouting vessels were guided towards the VEGF gradient. Furthermore, due to higher VEGF concentrations near the tumor, a dense branching microvascular network formed in these regions. At the beginning of the simulation, sprouts from a main vessel would tend to grow parallel to each other. Once capillary sprouts reached a certain distance from the main vessel, they could bend toward each other and ultimately join together in a known process called anastomoses. Tumor growth was exponential and rates were proportional to the number of CCs present (doubling time of 15.0 d, *R*^2^ = 0.98), Fig. 1B. This was consistent with the MDA-MB-231 cell line and growth patterns in mice^21^. In reality, nutrient supply depends on microvascular network characteristics like vessel length. The production of nutrients in our simulations was assumed to be proportional to the number of vessel nodes. Therefore, microvascular network connectivity and nutrient depletion produced more realistic tumor growth and morphology (see Fig. 1C). New microvessel branches and sprouts occurred in response to VEGF gradients and observed to grow towards hypoxic regions for nutrient supply. This subsequently caused time-dependent increases in microvascular density as illustrated in Fig. 1D.

**Figure 2:**
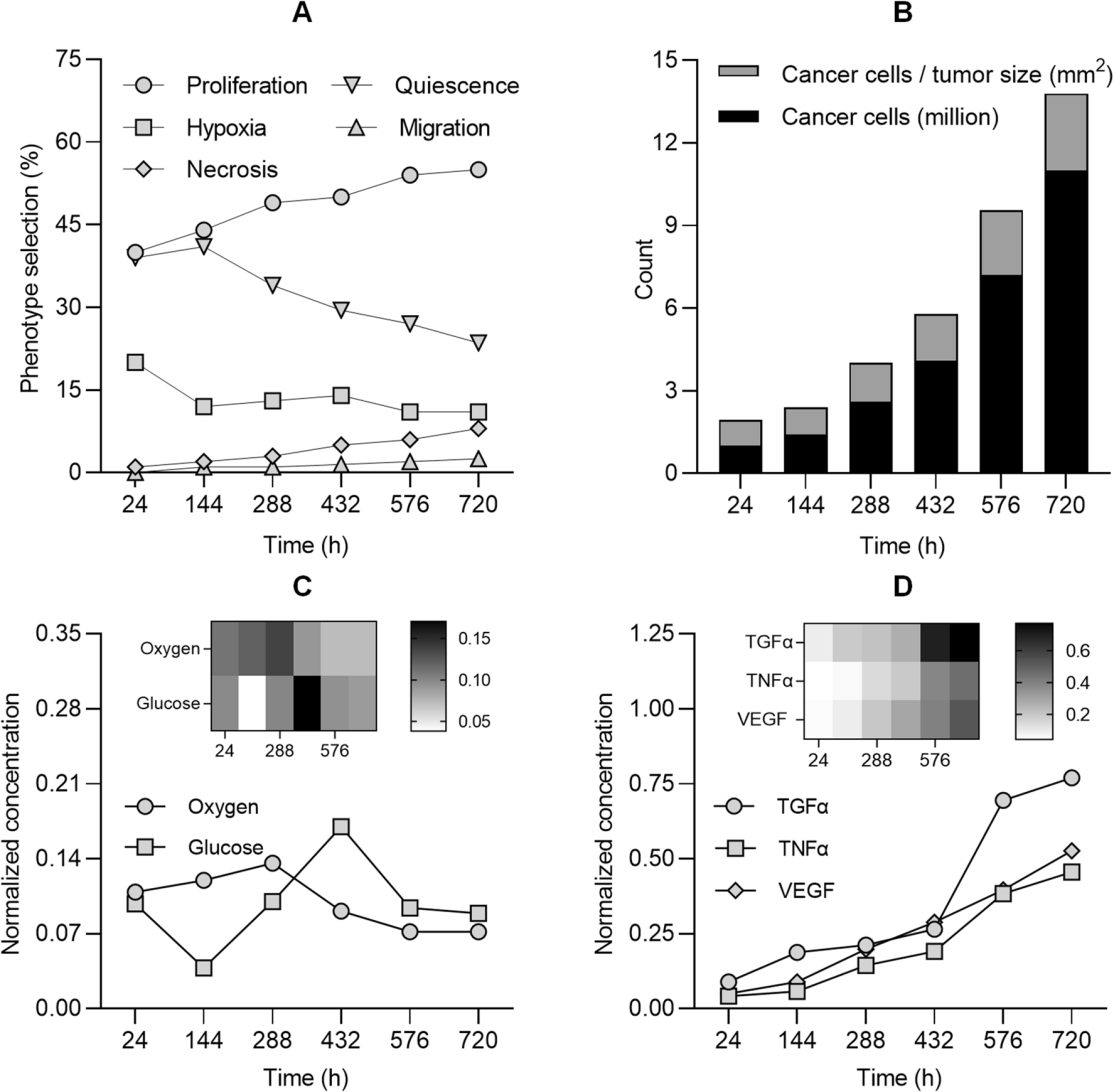
(**A**) Dynamic behavior and cancer cell phenotype selection during the test phase of the multiscale computational model. (**B**) Cumulative number of new cancer cells relative to tumor size during the test phase. Additional nutrients are supplied by the new microvascular network that further promotes tumor growth. Changing (**C**) oxygen and glucose concentrations and (**D**) transforming growth factor alpha (TGFα), tumor necrosis factor alpha (TNFα), and VEGF concentrations within the tumor microenvironment (TME).

Cells and vessels can be regarded as discrete entities (also referred to as agents) that can predict their phenotypes (i.e., actions) autonomously. This implies they act on their own without explicit direction in response to situations they encounter. The dynamic behavior of a tumor and the TME governing cancer growth during the test phase are detailed in Fig. 2A. In the training phase, as the TME changes at different scales following model rules, agents will respond to these changes by altering their action accordingly. This behavioral data is generated, stored, and then used for prediction during the test phase. One distinction between cancer and normal cells is that the later display density-dependent inhibition of proliferation^22^. As shown in Fig. 2B, model behavior dictates that CCs continue to proliferate independent of cell density. Furthermore**,** tumor size and the corresponding number of CCs on different days provides information about the tumor growth behavior. Once CCs stimulate microvessel growth by VEGF signaling, new microvessels nourish the growing cancer leading to increased cancer size. Following, microvascular sprouts were created after accumulation of CCs, which were recruited from the parent vessels. This programmed sequence of events, which controls sprouting in microvascular branch development, results in the tumor being increasingly penetrated by capillary vessels. Model behavior dictated that both oxygen and glucose concentrations remained relatively stable throughout the simulation period, Fig. 2C. Microvascular networks with shorter than average vessel lengths produced larger cancers and led to reduced nutrient concentration around the tumor periphery. As the difference between expansion of the microvascular network and cell population increased, nutrient concentration increased too and CCs were instructed to favor proliferation and migration with a greater probability compared to other phenotypes (see Fig. 2A). Furthermore, when transforming growth factor (TGFα) or tumor necrosis factor (TNFα) reaches neighboring cells, they are simulated to select proliferation/migration or necrosis phenotypes, respectively. As tumors grew larger, some CCs (especially in the mass center) got further away from the microvessels and cells were more likely to select a state of quiescence, necrosis or hypoxia. Upon inspection of Fig. 2D, it can be deduced that all prediction-based simulation results produced increased VEGF, TGFα, and TNFα concentrations, which noticeably impacted tumor growth patterns. As an example, for TGFα, we can summarize model behavior as follows. During the training phase, the more TGFα each agent has access to, the more probable it selected proliferation (based on DRL) and the agent earns an acceptable amount of reward. At the test phase, if the agent encountered conditions like a high amount of TGFα, it was more probable to predict proliferation as this decision comes from the collected dataset constructed during the training phase.

**Figure 3:**
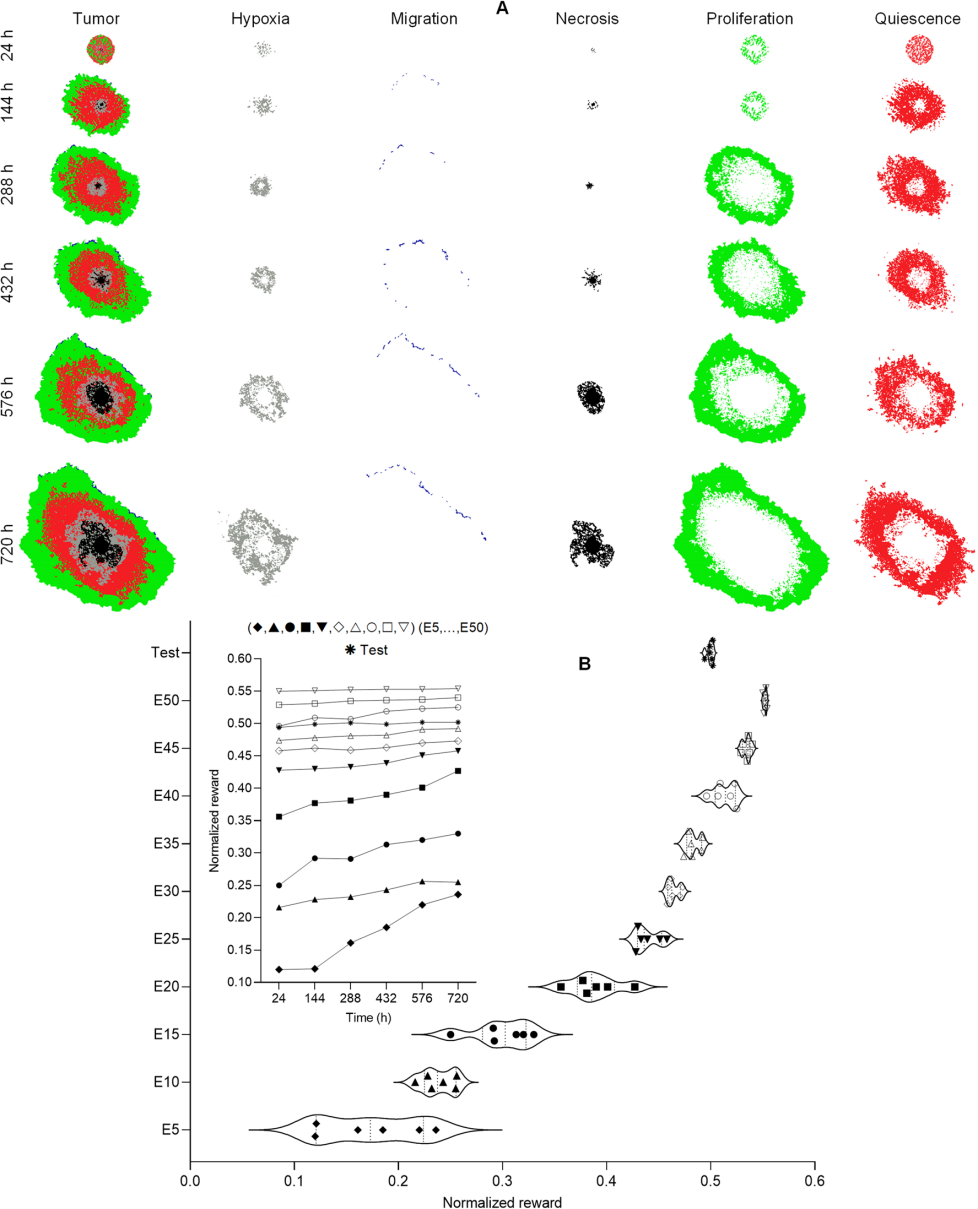
(**A**) Depiction of the simulated tumor environment and most commonly selected phenotype during the test phase. As the tumor grows, oxygen/glucose deficiency in the tumor center tends to change cell phenotype to states of hypoxia and necrosis. However, peripheral cancer cells proliferate and tumor growth continues throughout the 30-d modeling period. After time, a portion of the quiescent cells die and a necrotic core is generated at the tumor center. Subsequently, hypoxic cells release VEGF to stimulate nearby vessels to initiate sprouting and new microvascular growth. (**B**) Reward score curves illustrate the deep reinforcement learning (DRL) agent average reward score in both the simulation training and testing phases per 50 training episodes. An increasing reward score indicates that knowledge learned by the agents during each of the episodes is useful when learning the next episode.

Significant oxygen and glucose depletion by CCs leads to variable concentrations through the TMEs. Eventually, a deficiency leads to an upregulation of VEGF production that stimulates angiogenesis and new vessel growth. This new tumor condition is then used to update nutrient concentrations. Depicted in Fig. 3A, as the tumor grows due to nutrient deficiency, cells prefer to predict hypoxia or necrosis phenotypes in the test phase. With reduced nutrient concentration available at the tumor center, CCs have a greater chance of selecting necrosis versus hypoxia. Hypoxia is a state that indicates proliferation of CCs is occurring at a much higher rate compared to necrosis^23^. Additional cell proliferation around the tumor leads to enhancement of the existing microvascular network through angiogenic activity. Since CCs had a limited number of divisions, when the number of cell divisions exceeded an imposed cell division threshold, the probability of quiescence increased^24^. Further, physical challenges between tumor peripheral and interior CCs prohibited interior cell counts from increasing. As a result of these conditions, a significant portion of inner cells tended to select the quiescent phenotype. Greater proliferation rates of CCs cause local crowding, increased density, resource limitations, and further cell stimulation at the tumor periphery to select the migration phenotype. As described in the methodology section, the goal of agents during the computational model training phase were to maximize rewards they received by rewarding functions that make agents learn favorable policies. The training phase underwent 50 repetitions by passing the dataset between repetitions. As shown in Fig. 3B**,** the average reward score increases and sufficiently converged during the set number of training repetitions (i.e., upper limit). After training was complete, reward score measurements obtained during the test phase revealed test agents reached an optimal reward more quickly than in earlier repetitions of the training phase due to influence from the constructed training phase datasets. Further, our model reached a better reward score in the test phase by having the training repetition.

**Figure 4:**
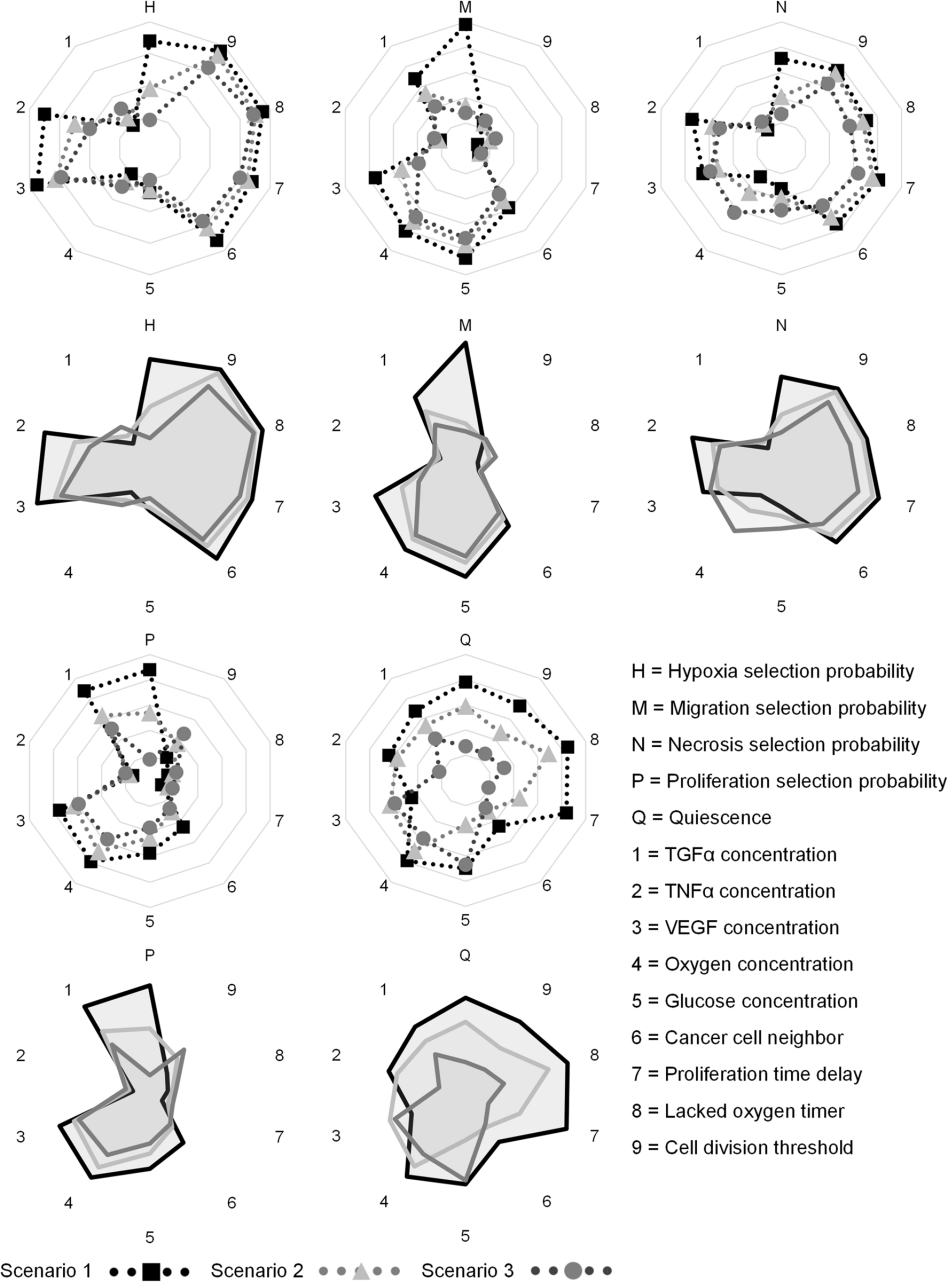
Relationship between phenotype probability selection and model parameters for three different scenarios used during the test phase: (1) phenotype selection probability < 33.3%, (2) 33.3% ≤ phenotype selection probability < 66.6%, (3) phenotype selection probability ≥ 66.6%.

Currently, there is a scarcity of scientific literature describing the quantitative relationship between phenotype selection probability of CCs and material concentration. For each test phase episode, therefore, the ratio of each predicted phenotype (e.g., cell proliferation) to other predicted phenotypes were obtained and then mapped to three predefined intervals based on their values. The average of all episode ratio values in each interval was calculated and finally average values for those three intervals were obtained and called the phenotype selection probability. Three scenarios were considered to assess the relationship between phenotype and diffusible factors (oxygen, glucose, TGFα, TNFα, and VEGF). Scenarios 1, 2, and 3 corresponded to cases in which the phenotype selection probability was less than 33.3%, between 33 and 66%, and greater than 66.6%, respectively. The goal of this scenario analysis was to study the predicted behavior of the tumor given uncertain environmental conditions. According to Fig. 4, findings were in good agreement with biological tumor behavior rules (see Table 2 for more details). As example, for proliferation in scenario 1 in which selection probability was greater than 66.6%, glucose, oxygen, and TGFα concentrations had a direct relationship with the proliferation selection probability, though the division threshold of CCs had an inverse relationship with selection probability.

The validity of the multiscale computational model was evaluated by independent mathematical and experimental considerations. Three simplified mathematical models were used to independently predict select features of tumor growth, including total count of CCs, mean microvascular length, and total number of microvascular branching points. When compared to the time-dependent mathematical descriptions for the number of CCs, average microvascular length, and number of microvascular branching points, the multiscale computational model produced similar trends and results, ${R}^{2}=0.87$, ${R}^{2}=0.99$, and ${R}^{2}=0.93 \left(p<0.001\right)$, respectively.

**Figure 5:**
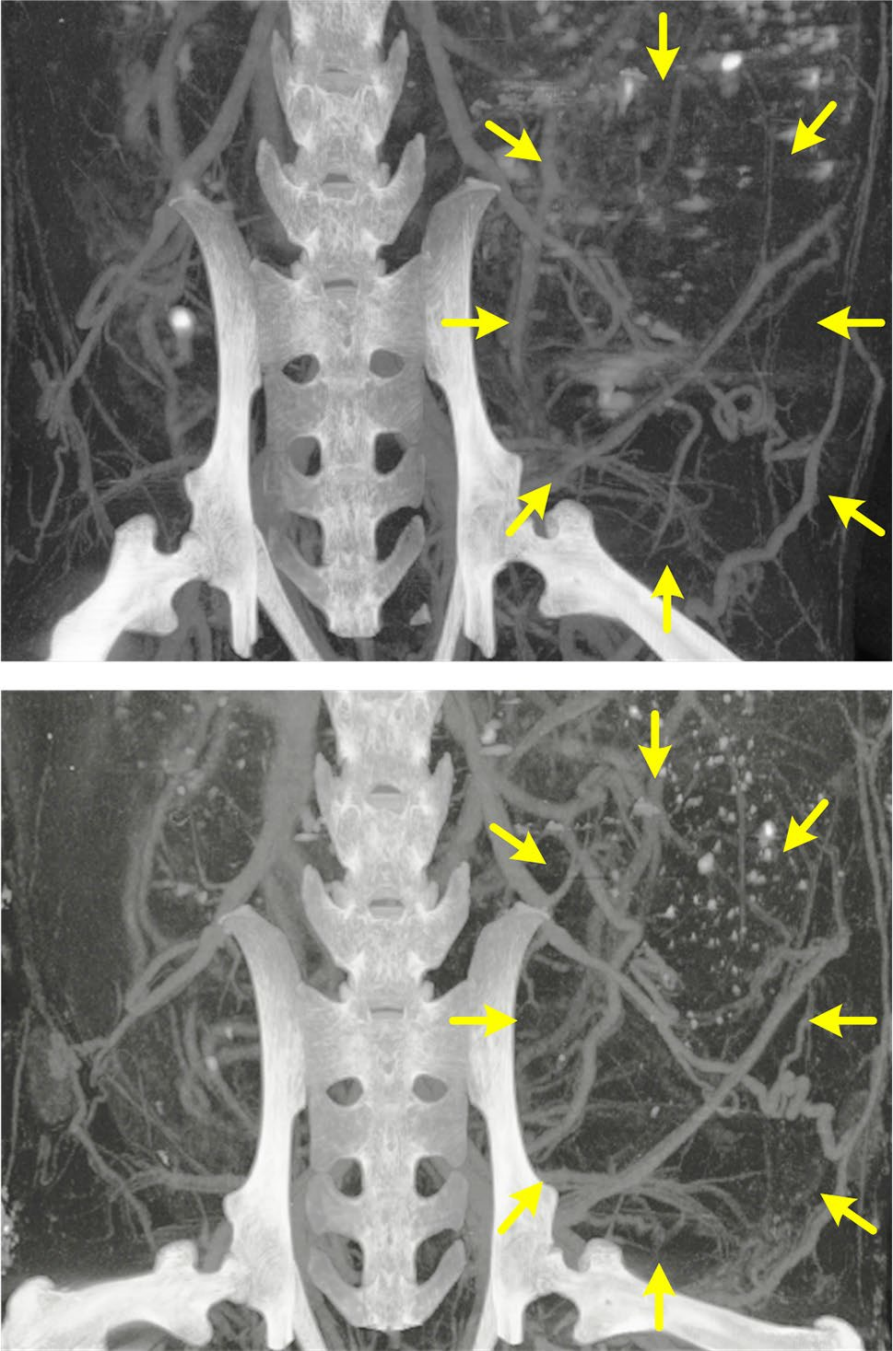
Series of preclinical in vivo contrast-enhanced microscale computed tomography (microCT) images of an aggressive breast cancer (yellow arrows) acquired in the same animal at reference baseline (top) and 2 wks (bottom).

While simulations can provide insight into how microvascular networks may develop, the most conclusive validation method is the use of actual experimental data that captures tumor and microvasculature growth over a specific time period of observation. To determine accuracy of the predicted tumor size, data was collected from two mice at distinct time points. Preclinical in vivo microCT images of an aggressive breast cancer acquired in the same animal at reference baseline and 2 wks thereafter are presented in Fig. 5. These images highlight microvascular detail of the growing tumor, which were used as input features to the multiscale computational model. Here we used longitudinal data from this one subject for the prediction-based simulation and to discover the variable input to output mapping by reward maximization. The prediction-based simulation was then validated by using data from an untested second subject by making predictions on the constructed dataset. In short, simulated results were very similar to those derived from experimental images not used during the training process and otherwise previously unseen. More specifically, actual tumor measurements at 6 and 7 wks were 1.52 and 1.64 cm^2^, respectively. Similarly, relative growth and simulated tumor size over a matched 7-d period were found to be 1.49 and 1.78 cm^2^. Differences were attributed to the inherent stochasticity of the model processes (rather than a deterministic form).

## Discussion

Computational models are increasingly being used for cancer forecasting^25^. Although several models have been detailed in the scientific literature, actual biological conditions are far more complex to the point that current computational models are not able to sufficiently describe all details^26^. In addition, such models can be computationally expensive introducing a trade-off between complexity and speed when incorporating clinical or experimental data^27^. With complex modeling problems, associated challenges that may arise include estimation of model parameters, availability of pertinent experimental data, model validation, and variable selection for model inclusions^28^.

The main focus of this paper was on the coincident evolution and morphology of microvascular networks and breast cancer growth in a preclinical setting. To limit the complexity of the model and to primarily focus on tumor modeling, processes like tumor morphology in volume space and several other biological and biochemical mechanisms including DNA damage/repair and genetic mechanisms were not incorporated. Our simplified 2-D computational model used experimentally-derived high-resolution microCT images of a tumor angiogenic network to initialize the microvascular network in simulation. The theories, assumptions, and parameter values were obtained from the literature detailing theoretical and experimental biology. This model incorporated three relevant biological scales at the microscopic, mesoscopic, macroscopic levels. At the microscopic scale, a system of partial differential equations (PDEs) simulated the dynamics of select signaling pathways. An automatic cell and vessel phenotype prediction procedure was used in the mesoscopic scale. Angiogenesis was coupled with tumor growth through VEGF secretion by the CCs at this scale. We employed PDEs to simulate the concentration changes of five extracellular chemical cues at the macroscopic scale (i.e., glucose, oxygen, TGFα, TNFα, and VEGF).

A comparison of results from model predictions showed that incorporating microCT imaging data with the prediction-based simulation led to an adequate agreement between the model and test image-derived data measurements. This highlights the importance of longitudinal data to improve the accuracy of cancer modeling^29^. Repeat and registered microCT images of breast cancer-bearing mice at different time points allows assessment of tumor features including cancer size and cell density. It was shown possible that a hybrid multiscale computational model could be designed that integrates different modeling techniques based on in vivo data, and implies that the model had flexibility to simulate fundamental cancer biological processes at an acceptable level of approximation. Model behavior was independently validated by theoretical considerations and experimental data. In short, simulated tumor growth patterns agreed with validation data not used during model development.

The flexibility of the multiscale model domain is expected to play a role in predicting the spatiotemporal behavior of tumor response to anticancer treatment. Such research will form the basis for future work. Obtaining an exact match between model and experimental data at later time points should not be expected, since we are modeling an extremely complex system characterized by an array of uncertainties. However, use of additional time points and medical images would allow the model to have a more complete dataset for comparison and provide opportunities for model refinement (recalibration), allowing the initial arbitrary step to be a baseline measurement and use of the remaining time points for tumor growth comparison, microvessel density quantification and model calibration. This approach should improve model results and provide more accurate tumor forecasts. Additionally, we plan to extend the model to volume space to simulate a more realistic assessment of in vivo tumor growth and heterogeneity with a focus on clinical studies. The potential impact of our newly developed multiscale computation model of tumor growth will further increase with the incorporation of additional realistic biological and physical features including tumor perfusion and interstitial pressure measurements at the microvascular level^4^, which can also be produced using noninvasive medical imaging-based techniques currently in various phases of technology development^30–34^.

## Methods

### Animal preparation

All procedures were carried out in accordance with relevant guidelines and regulations. Animal experiments were performed based on a protocol approved by the Institutional Animal Care and Use Committee (IACUC) at the University of Texas at Dallas. This manuscript complies with the ARRIVE guidelines for reporting animal research^35^. Six-wk-old female athymic nude mice (*N* = 2; Charles River Laboratories, Wilmington, MA) were implanted subcutaneously with two million breast cancer cells (MDA-MB-231, American Type Culture Collection, Manassas, VA) in the inguinal region of the mammary fat pad. The implanted tumors were allowed to grow for six weeks. Note that one animal was used for mathematical model training whereby the second animal was used for testing.

### Imaging protocol

Animals were anesthetized with 1 to 2% isoflurane in oxygen (V3000PK, Parkland Scientific, Coral Springs, FL) and placed on a temperature-controlled heating pad to maintain core levels (Rodent Surgical Monitor, AnimaLab, Poznan, Poland). A catheter was placed and secured in the tail vein before administration of a nanoparticulate contrast agent optimized for microCT angiography (200 µL; ExiTron nano 12000, Miltenyi Biotec, Bergisch Gladbach, Germany). This contrast agent has a long blood half-life enabling visualization of fine microvascular structures. After dosing, animals were transferred to a full body heated cradle and imaged using an ultra-high-resolution microCT system (OI/CT, MILabs, Utrecht, Netherlands). Exposure settings were 50 kV, 0.21 mA, 75 ms exposure time, and 360º rotation in 0.25º steps for a total of 1440 projections. Images were reconstructed using vendor software (PMOD Technologies LLC, Zurich, Switzerland) to a cubic voxel size of 20 μm, before saving as a DICOM format. DICOM files were then imported into OsiriX image analysis software (Pimeo, Bernex, Switzerland) for post-processing. The tumor microvascular network (contrast agent) was highlighted by rendering with a bandpass filter of 500 to 1000 Hounsfield Units (HU) (see Fig. 5). After global selection of the entire tumor space, a K-means clustering method was used to determine a topological structure of the microvascular network and to partition the microCT image into two mutually exclusive clusters (i.e., dark background and bright target blood vessel pixels)^36^. This automatic unsupervised vessel classification algorithm segmented the entire microCT image to isolate the microvascular network supporting tumor growth. These segmented images were then used as inputs to a multiscale mathematical model to forecast tumor growth.

### Multiscale computational model of tumor growth

A mathematical model was developed to simulate the spatiotemporal dynamics of tumor growth in vascularized tissue by PDEs, as well as DRL and agent-based modeling (ABM). This simulation performed model calculations at the microscope, mesoscopic, and macroscopic scale. Therefore, the model consists of two components, a continuous (PDE) and discrete element (ABM and DRL). The discrete components are used to describe the evolution of the cells and vessels. On the other hand, the continuous components are used to describe the environment of the tumor. The model environment was formulated on a regular grid that subdivides the simulation domain into distinct lattice sites called a tumor microenvironments (TME). During tumor growth, the main regulatory substances that were considered include oxygen, glucose, TGFα, TNFα, and VEGF, that were collectively referred to as diffusible factors. With cells and vessels, diffusible factors control TME dynamics. The number of diffusible factors for all TMEs were assumed uniform at initialization and during simulation new levels were calculated according to the consumption or production rate of cells and vessels placed in each TME. At each simulation time step, the TME had a homogeneous concentration of diffusible factors which implied all cells or vessels within an individual TME are equally exposed. Multiple cells can be placed in each TME, according to a predefined carrying capacity (i.e., maximum number of cells located at each TME). Note there was no limitation on the number of vessels inside each TME. The concept of a Moor neighborhood was used to allow cell movement when a migration phenotype was selected. TMEs represent possible locations where cells or vessels may reside, with the advantage that this type of subdivided grid is capable of containing heterogeneous populations with distinct TME characteristic associated to each cell or vessel within. At the microscopic scale, two important signaling pathways including the tumor necrosis factor receptor (TNFR) and epidermal growth factor receptor (EGFR) were simulated. This enabled agents to respond and adapt to their environment, as they need to receive and process information (or signals) that originate outside the cell^37,38^. These two signals affect cell decisions during phenotype prediction since values are two attributes of a dataset used by the proposed DRL method. At the mesoscopic scale, cell and vessel phenotypes were assigned, which included proliferation, quiescence, hypoxia, necrosis and migration for cells, and quiescence, branch points and sprouting for vessels. The tumor was treated as a mass of agents (cells) and the angiogenic process was dependent on reinforcement modeling. Finally, at the macroscopic scale, a PDE equation describing diffusion was used to determine diffusible factor transport inside each TME and for calculation of oxygen and glucose consumption and production of TGFα and TNFα by cells. During the angiogenic phase, hypoxic and necrotic cells secreted VEGF into the TME and also based on PDE solutions.

**Figure 6:**
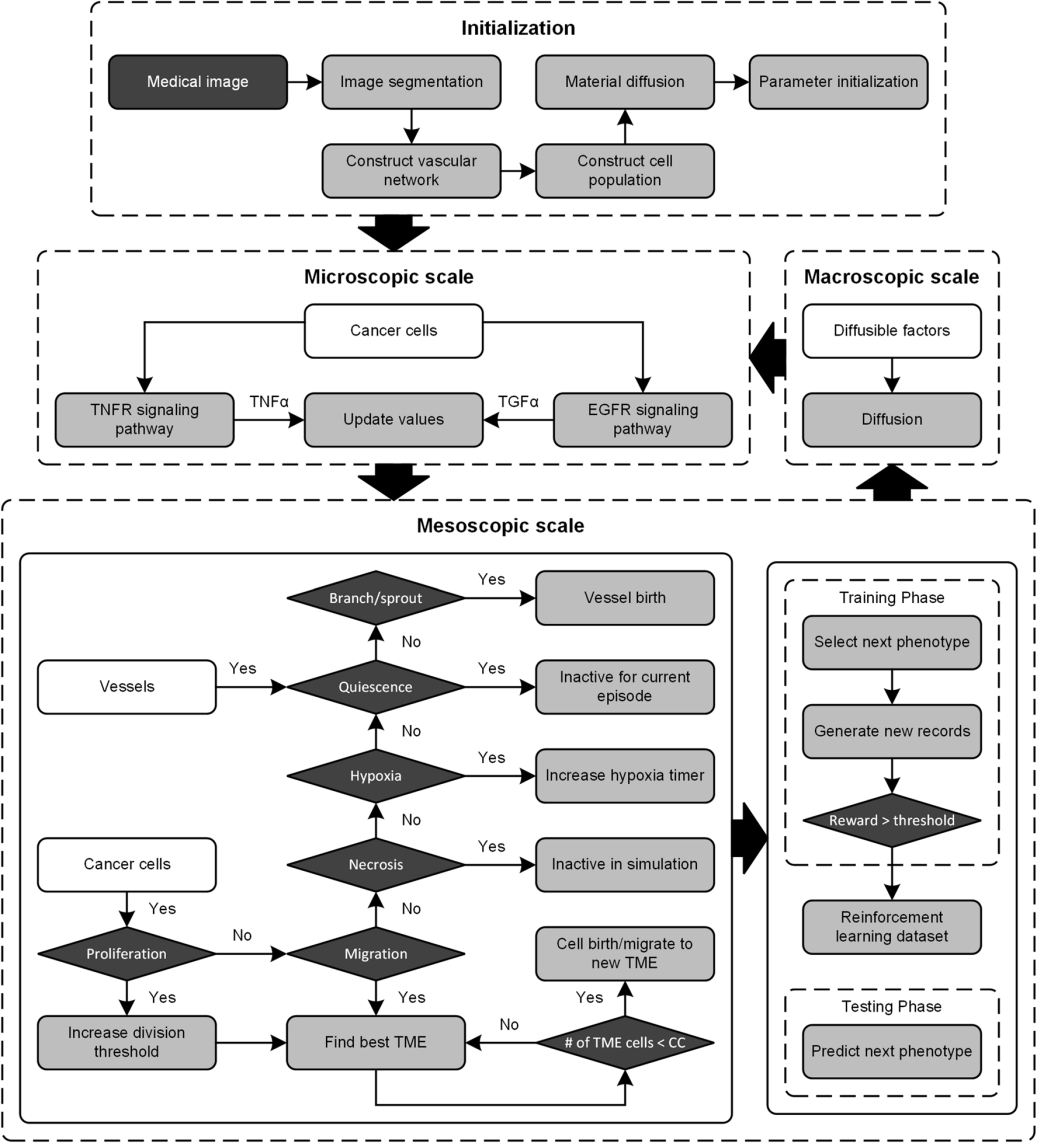
Flow chart and decision tree detailing the multiscale computational model for predicting tumor growth using input from information contained in medical images, including tumor location and microvascular network shape. CC Carrying capacity, EGFR epidermal growth factor receptor, TNFR tumor necrosis factor receptor.

After initializing model parameters, the multiscale process was repeated for each episode until the simulation ended, which was bound by a fixed number of episodes (i.e., time steps). The objective of each episode (that has three scales) was to better exploit the situated possibilities for data transfer amongst the agents in the model, whereas the objective of the simulation (repeat episodes) was to achieve maximum coherence across all episodes. A summary of the multiscale computational modeling process is depicted in Fig. 6 and valid for both the training and testing phases. The model is briefly summarized as follows:

- Step 1: Extract relevant tumor and microvascular features from a contrast-enhanced microCT image and use to initialize a spatial domain (e.g., cells, diffusible nutrients, etc.) and model tumor growth.
- Step 2: Run the microscopic scale.
  *For training and testing phases:* Execute the EGFR/TNFR signaling pathways and refine TGFα/TNFα concentrations.
- Step 3: Run mesoscopic scale.
  *For training phase*: During current cell and vessel phenotype execution, calculate quality values (known as Q-values) for all phenotypes based on DRL policies and select the next phenotype based on a weighted random selection (to have better exploration). Observe environment data and add to a dataset if reward of next phenotype is more than a predefined threshold.
  *For testing phase*: For current cell and vessel phenotype execution, predict next phenotype based on neural network.
- Step 4: Run macroscopic scale:
  *For training and testing phases*: Refine the environment by diffusing oxygen, glucose, VEGF, TGFα, and TNFα concentration.
- Step 5: Repeat Steps 2 to 4 until simulation ends.

**Figure 7:**
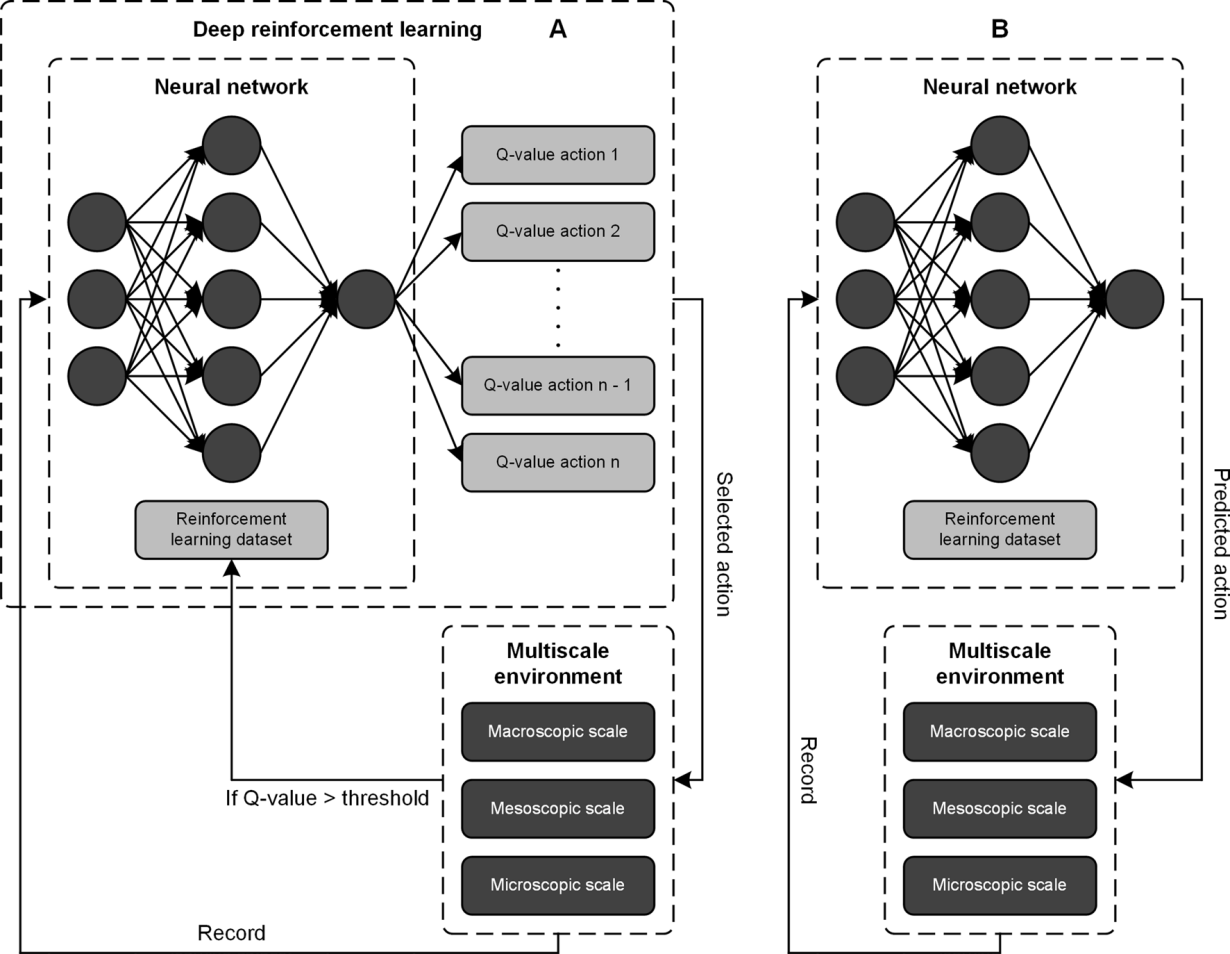
Flow chart detailing the deep reinforcement learning model for training and testing on patterns generated by the multiscale simulation for predicting tumor growth using input from medical image-derived information.

Note the multiscale computational model has both a training and testing phase. In the training phase a DRL dataset is generated (Fig. 7A) while in the testing phase all agents used this dataset to predict a preferred action due to changes in the TME (Fig. 7B). Follows is a more detailed description of the model including governing equations and assumptions.

### Model domain, geometry, and initialization

During model training and testing phases, the geometry of the initial microvascular network was derived from a segmented in vivo microCT image obtained from breast cancer-bearing mice. An interesting component of the multiscale computational model is that it initializes with a microCT image-derived features of the tumor environment rather than a more randomized beginning. Given a microCT image and the segmented tumor space, an initial diffusible factor diffusion ${C}_{M}^{0}$ (at time $t=0$) was specified using a normal distribution for each TME according to the following equation:

1 $${C}_{M}^{0} = \left\{\begin{array}{ll}{\overline{C} }_{M}+\left({\widehat{C}}_{M}-{\overline{C} }_{M}\right){e}^{\frac{-2{d}_{TME} }{{\sigma }^{2}}}& M=\left\{\text{glucose, oxygen}\right\}\\ {\overline{C} }_{M}{e}^{\frac{-2{D}_{M}}{{\sigma }^{2}}}& M=\left\{\text{TGF}\alpha ,\text{TNF}\alpha \right\}\end{array}\right.$$

where ${d}_{TME}$ is the average distance between cells and vessels inside the TME, ${\overline{C} }_{M}$ the normal material concentration (µM), ${\widehat{C}}_{M}$ the maximum material concentration (µM), ${D}_{M}$ the material diffusion coefficient (${\text{cm}}^{2}{\text{ s}}^{-1}$), and ${\sigma }^{2}$ the variance of the distribution function (set to 0.3). A summary of model parameters and value assignments are found in Table 1.

**Table 1:** Summary of select multiscale computational model parameters and value assignments using during simulation.

	Oxygen	Glucose	TNFα	TGFα	VEGF	Refs
${\overline{C} }_{M}$ [μM]	34.8	1.7 × 10^4^	6.0 × 10^–3^	2.0 × 10^–3^	–	^55^
${\widehat{C}}_{M}$ [μM]	55.6	5.7 × 10^4^	1.0 × 10^–6^	90.1	–	^41,52,55^
${D}_{M}$[${\text{cm}}^{2} {\text{s}}^{-1}$]	1.67 × 10^–7^	6.7 × 10^–7^	1.5 × 10^–7^	5.12 × 10^–7^	2.9 × 10^–7^	^49,55,56^
${\rho }_{M}$ [$\text{cm }{\text{s}}^{-1}$]	10	3.0 × 10^3^	0.82	5.5 × 10^–2^	1.0 × 10^3^	^20,49^

### Microscopic scale

The microscopic scale describes molecular and subcellular phenomena, which includes cell signaling cascades. A signaling pathway was a significant component because cell fate is determined by signals received from the TME. On this scale, PDEs are commonly used and parameter calculations of various signaling pathways are performed at the mesoscopic and macroscopic scales^39^. Two important signaling pathways, namely, EGFR and TNFR, were implemented with use of PDEs as these signals increase TGFα and TNFα concentrations, respectively^40,41^. Within a TME at each time step, EGFR and TNFR signaling pathways begin with binding of the ligands TGFα/TNFα to EGFR/TNFR (see Supplementary Information). This impacted cellular decisions on migration, proliferation/necrosis, or quiescence, throughout the training phase according to rules defined in Table 2.

**Table 2:** Policies for the multiscale computational model used during simulation, which are functional map states and probability distributions for possible actions.

No.	Policy description
1	If oxygen concentration is (higher)/(lower) than a threshold value (1.175), the chance of selecting (proliferation and migration)/(hypoxia and necrosis) will increase^50^
2	If glucose concentration is (higher)/(between)/(lower) than an (active)/(active and dead)/(dead) threshold, the chance of selecting (proliferation and migration)/(quiescence )/(necrosis) increases (active threshold = 16, dead threshold = 8)^52^
3	If TNFα concentration is (higher)/(lower) than a threshold value (0.6), the chance of selecting (necrosis)/(quiescence) increases^41^
4	If TGFα concentration is (higher)/(lower) than a threshold value (0.6), the chance of selecting (proliferation)/(migration) increases^52^
5	If VEGF concentration is (higher)/(lower) than a threshold value (0.3), the chance of selecting (sprout)/(quiescence) increases^57^
6	If vessel cell age is (higher)/(lower) than a threshold value (18), the chance of selecting (branch)/(quiescence) increases^39^
7	If division counter is (higher)/(lower) than a threshold value (50), the chance of selecting (quiescence)/(proliferation) increases^24^
8	If proliferation time delay is (higher)/(lower) than a threshold value (4), the chance of selecting (hypoxia)/(quiescence) increases^58^
9	If oxygen deficiency timer is (higher)/(lower) than a threshold value (67), the chance of selecting (necrosis)/(hypoxia) increases^44^
10	The more cancerous cell neighbors a CC has, the more likely it is to migrate^58^

The goal of the agent is to optimize future rewards and, specifically, to find a policy that maximize reward.

### Deep reinforcement learning

Reinforcement learning is a particular area of machine learning concerned with how intelligent agents should select actions in order to maximize a prescribed reward through trial-and-error interactions within a dynamic environment^42^. DRL is the combination of reinforcement learning and deep learning to make these trial-and-error decisions^43^. At the mesoscopic scale detailed below, model agents seek to have a desired phenotype at each simulation time step. For DRL agents to achieve a desired phenotype during the training phase, it must first explore what is possible within the TME and what constitutes progress towards that phenotype. For the multiscale computational model summarized in Figs. 6 and 7, DRL is a method whereby agents become intelligent and create new, optimal behaviors based on prescribed rewards and TME state.

### Agent-based modeling

ABM is a powerful approach that approximates cells as isotropic and autonomous entities. One of the promises of ABM is the potential to have agents make decisions in dynamic environments. Typical ABM functionality is based on understanding the influence of multiple agents that interact with each other in any given TME. Agents react to specific stimuli based on defined rules, but they don't learn how to act intelligently. In our model, a DRL algorithm was incorporated into an ABM framework. The DRL uses the ABM for constructing a dataset based on observational data and reward generation, while the ABM uses the DRL to predict the next agent phenotype. At the mesoscopic level, each cell or vessel is an independent agent that can take an action (phenotype) based on TME observations. By taking an action, the agent changes state and causes consumption or production of some diffusible factors at the macroscopic scale.

### Mesoscopic scale

At this intermediate tissue level, cells and vessels are represented as individual entities (agents) that have their own phenotype, and can predict their phenotype to have an acute response to external or internal stimuli like lack of oxygen. Vascular endothelial agents with tip and stalk differentiation were used to implement angiogenesis in simulation. Tip cells regulated new microvessel formation and could undergo branching, sprouting, or quiescence, whereby stalk cells could only undergo sprouting or quiescence. If a tip vessel sprouted, it was assumed they elongated at a rate of 1 µm min^–1^ (60 µm after one episode), which was biased towards TME selection^44^. The selected TME was from the current and Moore neighboring TMEs that had the maximum VEGF concentration. The destination point was a random location inside the selected TME. By calculating the distance between the origin and the destination points, the required time to reach the destination was determined (i.e., number of episodes). Tip cells did not change sprout phenotype until reaching the destination point. During episodes of sprout processing, a tip cell moved forward and a new stalk cell was created at the previous location. Both vessels and cells could enter a dormant state. This quiescence phenotype was characterized by less nutrient consumption.

Cancer cells were only allowed to proliferate if the number of CCs in a TME was less than the defined carrying capacity. If space was available, new cells were randomly located in the selected TME. The selected TME was the one among all candidates that had the maximum nutrient concentration. If there was no space available for both offspring cells, a proliferation time delay (attribute of the dataset) increased. Cells migrated along the environment to available TMEs (in terms of space) belonging to the Moore neighborhood with the highest nutrient concentration. The migration rate of tumor cells was assumed to be 0.75 μm/min (45 μm after one episode) and cell did not change migration phenotype until reaching the destination point^45^. Each cell had a property called the oxygen deficiency timer (another attribute of the dataset). This value was increased when a cell selected a hypoxic or necrotic phenotype. During the multiscale computational model training phase, agents interacted with the TME and other agents to create a list of transitions and action outcomes based on DRL. For the ABM, agents were designed to maximize a reward based on predefined rules and the DRL algorithm. The basic rules (or model policies) governing microvessel growth and patterning are defined in Table 2.

Using an incremental model-free reinforcement learning algorithm and a slight variation of the popular Q-learning algorithm, a state-action-reward-state-action (SARSA) algorithm^46^ produced a model of cell behavior and simulation of tumor growth. Since there was no final state for cell decisions, an incremental approach whereby learning takes place at any time was appropriate. Furthermore, there was no behavioral model for environmental agents deciding on actions for each state, so it was assumed a model-free approach was helpful. Consequently, SARSA could be considered one of the better options for decision making. A SARSA agent interacts with the TME and updates the policy based on the phenotype (action) taken. An updated Q-value in this case represented how useful a given action was in gaining some future reward ${r}_{t+1}$ as follows:

2 $$Q\left({s}_{t},{a}_{t}\right)=\left(1-\alpha \right)Q\left({s}_{t},{a}_{t}\right)+\alpha \left[{r}_{t+1}+ \gamma Q\left({s}_{t+1},{a}_{t+1}\right)\right]$$

where

3 $${r}_{t+1}=\sum_{\text{for each policy}}\text{tanh}\left(x-{T}_{policy}\right)$$

and variable $x$ is a policy state value, $\alpha$ is learning rate, and $\gamma$ is a discount factor that specified the time delay for any future rewards. SARSA learns the Q-values associated with taking the policy it follows, which depended on the current agent state (${s}_{t}$), action the agent chooses (${a}_{t}$), state that the agent enters after taking an action (${s}_{t+1}$), next action the agent chooses in its new state (${a}_{t+1}$), and the final reward the agent receives (${r}_{t+1}$). For the first episode of the multiscale computational model (i.e., $t=0$) the simulation was initialized and each agent was assigned a random action (phenotype) before *Q* and *r* values were calculated for each agent and subsequent episode. Rewards are scalar values and calculated using Eq. (3) as a sum of difference measure between agent state variables and each policy threshold ${T}_{policy}$ (called a state-value function). Table 2 describes in more detail the model policies and the relationship between state variables and actions. In short, the state-value function for model policy estimates “how good” it is for the agent to be in for a given state (or defined in terms of future reward).

Datasets constructed during the training phase represented a record of attributes of all agents including cell variables (i.e., phenotype, Q-value, etc.) and TME variables (i.e., diffusible factor concentration, number of CCs and microvessels). For each agent in the training phase and all possible actions, Q-values were computed according to Eq. (2), and then an incremental record was added to the dataset. Incoming data was added to the dataset if the value of the current reward was greater than a predefined threshold. Note this incremental approach had a beneficial impact on computational performance. A weighted random selection was applied where each agent selected an action among all possible actions. The idea behind the weighted random selection of the actions was to total all Q-values (ΣQ), generate a random number between 0 and ΣQ, and then the selected action was one that falls in the associated range. This implied selection of a random action from all actions was from the probability of that action (or Q-value). This led to better exploration during the training phase. Here DRL had the ability to recognize patterns instead of mapping every state to the best action. During the model test phase and given a previously unseen microCT image as input data, a Waikato environment for knowledge analysis (WEKA)-based fully connected feedforward neural network (in lieu of computing Q-values) was used. This model component predicted a future action to take given the constructed dataset and current record (current/previous action, phenotype, and current/previous state variable values)^47^.

### Macroscopic scale

An important component of the multiscale computational model was the simulated release of material inside the TME. Each TME could be occupied by cells until it reached the population size limit (termed carrying capacity, $C$) and there was no constraint for vessel count within. Oxygen and glucose levels in the TME were supplied by a dynamic microvascular network subject to remodeling and additional growth (angiogenesis). All cells acted as proverbial sinks for oxygen and sources of VEGF, TGFα, and TNFα. Oxygen and glucose (i.e., nutrients) were supplied by the established microvascular network that acted as a sink for VEGF, TGFα, and TNFα, and source of oxygen and glucose.

The diffusion equation describing the spatiotemporal evolution of all material concentrations (including nutrients and VEGF) for each TME at time $t+1$ was:

4 $${C}_{M}^{t+1} ={C}_{M}^{t}+{\Delta tD}_{M}\Delta {C}_{M}^{t}{A}^{-1}+{S}_{M}^{t}-{U}_{M}^{t}-{W}_{M}^{t}$$

where ${C}_{M}^{t+1}$ is the material concentration (µM) at the next time step ($t+1$), $\Delta t$ the time difference between two episodes (set to 1 h), ${C}_{M}^{t}$ the local TME concentration (µM), $\Delta {C}_{M}^{t}$ the concentration difference between the eight Moore neighbors (µM), $A$ the TME area (cm^2^), ${S}_{M}^{t}$ the material secretion (µM), and ${U}_{M}^{t}$ and ${W}_{M}^{t}$ denotes material uptake and waste (µM), respectively. The sum $\Delta {C}_{M}^{t}$ of each difference in material concentration relative to a neighbor ${\acute{C}}_{M}^{t}$ and current material concentration for each time step is then calculated as follows:

5 $$\Delta {C}_{M}^{t} =\sum \left({\acute{C}}_{M}^{t}-{C}_{M}^{t}\right)$$

where $\acute{C}$ (µM) denotes concentration of the Moore neighbors. Oxygen and glucose production in each TME was dependent on microvascular radius ${R}^{t}$ (cm), permeability ${\rho }_{M}$ ($\text{cm }{\text{s}}^{-1}$), and concentration ${\zeta }_{M}^{t}$ (µM)^48–50^. VEGF production was then calculated by cell phenotype within each TME^51^. Inside each TME, secretion rates of both TGFα and TNFα were assumed constant and regulated by all cells:^52^

6 $${S}_{M}^{t} =\left\{\begin{array}{ll}{2\pi \Delta t{A}^{-1}R}^{t}{\rho }_{M}\left({\zeta }_{M}^{t}-{C}_{M}^{t}\right)& \text{for } M=\text{nutrient}\\ {10}^{-2}\times \sum_{\text{for all cells}}{S}_{VEGF}^{t}& \text{for } M=\text{VEGF}\\ \Delta t\sum_{\text{for all cells}}{\rho }_{M}& \text{for }M=\left\{\text{TGF}\alpha ,\text{TNF}\alpha \right\}\end{array}\right.$$

where

7 $${R}^{t} = \sum_{\text{for all vessels}}\left\{\begin{array}{ll}3\times {10}^{-3}& \text{for stalk cells passing through the TME}\\ 1\times {10}^{-3}& \text{for tip cells passing through the TME}\end{array}\right.$$

8 $${\zeta }_{M}^{t} =\sum_{\text{for all vessels}}\left\{\begin{array}{l}\text{if stalk cell passes through TME }\left\{\begin{array}{c}\begin{array}{cc}5.7\times {10}^{4}& \text{for } M=\text{glucose}\end{array}\\ \begin{array}{cc}5.6\times {10}^{1}& \text{for } M=\text{oxygen}\end{array}\end{array}\right.\\ \text{if tip cell passes through TME }\left\{\begin{array}{c}\begin{array}{cc}1.7\times {10}^{4}& \text{for } M=\text{glucose}\end{array}\\ \begin{array}{cc}4.2\times {10}^{1}& \text{for } M=\text{oxygen}\end{array}\end{array}\right.\end{array}\right.$$

and

9 $${S}_{VEGF}^{t}=\left\{\begin{array}{ll}{2}^{0}& \text{if phenotype is proliferation}\\ {2}^{1}& \text{if phenotype is migration}\\ {2}^{2}& \text{if phenotype is quiescence}\\ {2}^{3}& \text{if phenotype is hypoxia}\\ {2}^{4}& \text{if phenotype is necrosis}\\ 0& \text{if no cell exists in the TME}\end{array}\right.$$

For the vessel model, tip cells are present at the leading edge of the vessel and express lower nutrient levels than stalk cells that are positioned behind the tip cell. Both the VEGF secretion rate ${S}_{VEGF}^{t}$ (µM) and nutrient uptake rate ${U}_{N}^{t}$ (µM) for both cell types was dependent on cell phenotype. It was assumed TNFα and TGFα consumption was minimal and so not considered. However, consumption of VEGF in each TME relied on both microvascular size and VEGF permeability ${\rho }_{VEGF}$ ($\text{cm }{\text{s}}^{-1}$)^48–50^. Oxygen and glucose uptake is then determined by the cell phenotypes within each TME and concentration:

10 $${U}_{M}^{t}= \left\{\begin{array}{ll}0& M=\left\{\text{TGF}\alpha ,\text{TNF}\alpha \right\}\\ {2\pi \Delta t{A}^{-1}R}^{t}{\rho }_{M}{C}_{M}^{t}& M=\text{VEGF}\\ {{10}^{-3}\times C}_{M}^{t}\sum_{\text{for all cells}}{U}_{N}^{t}& M=\left\{\text{glucose},\text{ oxygen}\right\}\end{array}\right.$$

where nutrient uptake is found by:

11 $${U}_{N}^{t}=\left\{\begin{array}{ll}{2}^{4}& \text{if phenotype is proliferation}\\ {2}^{3}& \text{if phenotype is migration}\\ {2}^{2}& \text{if phenotype is quiescence}\\ {2}^{1}& \text{if phenotype is hypoxia}\\ {2}^{0}& \text{if phenotype is necrosis}\\ 0& \text{if no cell exists in the TME}\end{array}\right.$$

The VEGF decay rate was the last variable recorded and computed as follows:

12 $${W}_{M}^{t}=\sum_{\text{for all vessels}}\left\{\begin{array}{ll}{10}^{-2}\times \sum {C}_{M}^{t}& M=\text{VEGF}\\ 0& M=\text{all other material}\end{array}\right.$$

The decay rate for all other materials was considered insignificant and otherwise not modeled.

### Neural network for tumor growth prediction

Tumor growth predictions were accomplished using a neural network after generation of a training phase dataset. During the computational model testing phase, the neural network was used to predict a preferred action. The applied neural network consisted of three fully connected layers including the input, hidden, and an output layer that produced the output variables. The number of nodes in the input layer was equal to the number of cell and vessel attributes. Output layer nodes for the cells and vessels were five and three, respectively, and equal to the number of their phenotypes. There were two hidden layer levels with 35 and 25 nodes for both cells and vessels, respectively. After changing phenotype of each agent (i.e., cell or vessel) according to stated DRL policies, if the reward was more than threshold, a new record with defined attributes was added to the dataset. The datasets generated during the training phase consisted of the following attributes for a cell (vessel): Previous and current diffusible factor concentrations, number of cells (vessels) inside a cell (vessel) TME, previous and current division counter, number of cells that are necrotic and hypoxic (vessels that have branches and sprouts), cell (vessel) age and proliferation time delay, cell oxygen deficiency timer, previous and current cell (vessel) Q-values, previous and current cell (vessel) phenotypes, previous and current cell (vessel) neighbor phenotype. A dataset normalization process was conducted to rescale all attribute values to the range [0, 1]. By applying the neural network in the testing phase, agents can act autonomously and change phenotype without any external direction or policy.

### Model validation and performance measures

The validity of the multiscale computational model was assessed by mathematical and experimental considerations. These validation exercises provided an opportunity to examine the impact of the unobservable prediction processes, and accuracy of model predictions provided insight into the plausibility of different model assumptions. Three mathematical models were used to predict select temporal features of tumor growth throughout a 30-d simulation period, including total cancer cell count $n\left(t\right)$:

13 $$n\left(t\right)={n}_{0}{e}^{\frac{{\alpha }_{n}}{{\beta }_{n}}\left(1-{e}^{-{\beta }_{n}t}\right)}$$

mean microvascular length $g\left(t\right)$:

14 $$g\left(t\right)={g}_{0}+{\alpha }_{g}{\left(1+{e}^{-\frac{t-{t}_{1/2}}{{\beta }_{g}}}\right)}^{-1}$$

and total number of microvascular branching points $b\left(t\right)$:

15 $$b\left(t\right)={b}_{0}+{\alpha }_{b}{e}^{{\beta }_{b}t}$$

where $\alpha$ and $\beta$ are function shape parameters, ${t}_{1/2}$ is the simulation half-life (h), and ${n}_{0}$, ${g}_{0}$, and ${b}_{0}$ are the initial cancer cell population size, vessel lengths, and number of vessel branches, respectively^23,48,53,54^. From the literature, parameters for Eqs. (13) to (15) were chosen to be $\left({n}_{0}, {\alpha }_{n},{\beta }_{n}\right)=\left({10}^{6}, \text{0.09,0.01}\right)$, $\left({g}_{0}, {\alpha }_{g},{\beta }_{g},{t}_{1/2}\right)=\left(0, {58.5}^{3},\text{62,360}\right)$, and $\left({b}_{0}, {\alpha }_{b},{\beta }_{b}\right)=\left(0, \text{0.05,16}\right)$, respectively. Changes in tumor size and doubling time was assessed using a standard expression for exponential growth. Robustness of time-dependent tumor growth measurables found during simulation validation were compared to independent mathematical models using a Pearson correlation analysis. A *p*-value less than 0.05 was considered statistically significant. All analyses were performed using Prism 9.0 (GraphPad Software, San Diego, CA).

## Supplementary Information

Supplementary Information.
