# Supplementary material for: Multiscale computational modeling of cancer growth using features derived from microCT images
Source: Sci Rep. 2021 Sep 17;11:18524. doi: 10.1038/s41598-021-97966-1 (PMC8448838; doi:10.1038/s41598-021-97966-1)
Supplement: Supplementary file 1 — Supplementary Information. [file 41598_2021_97966_MOESM1_ESM.docx]

# Appendix I

**TNFR signaling pathways**: Summary of kinetic equations used during development of the multiscale computational model of tumor growth. TNF signaling was based on the following tables.

Table 1. Partial differential equations (PDEs) for TNFR calculations.^1^

| $\frac{dc_{1}}{dt} = -k_{1}. c_{1}. c_{2}+ k_{2}. c_{3}$ | $\frac{dc_{17}}{c_{t}}= -k_{15}. c_{9}. c_{17}+ k_{16}. c_{18}+ k_{20}. c_{21}$ |
| --- | --- |
| $\frac{dc_{2}}{dt} = -k_{1}. c_{1}. c_{2}+ k_{2}. c_{3}+ k_{17}. c_{18}+ k_{11}. c_{11}$ | $\frac{dc_{18}}{dt} = k_{15}. c_{9}. c_{17}. k_{16}. c_{18}. k_{17}. c_{18}$ |
| $\frac{dc_{3}}{dt} = k_{1}. c_{1}. c_{2}. k_{2}. c_{3}. k_{3}. c_{3}. c_{4}+ k_{4}. c_{5}$ | $\frac{dc_{19}}{c_{t}}= k_{17}. c_{18}. k_{18}. c_{19}. c_{20}+ k_{19}. c_{21}$ |
| $\frac{dc_{4}}{dt} = -k_{3}. c_{3}. c_{4}+ k_{4}. c_{5}+ k_{11}. c_{11}+ k_{20}. c_{21}$ | $\frac{dc_{20}}{dt} = -k_{18}. c_{19}. c_{20}+ k_{19}. c_{21}$ |
| $\frac{dc_{5}}{dt} = k_{3}. c_{3}. c_{4}. k_{4}. c_{5}. k_{5}. c_{5}. c_{6}+ k_{6}. c_{7}$ | $\frac{dc_{21}}{dt} = k_{18}. c_{19}. c_{20}. k_{19}. c_{21}. k_{20}. c_{21}$ |
| $\frac{dc_{6}}{dt} = -k_{5}. c_{5}. c_{6}+ k_{6}. c_{7}+ k_{11}. c_{11}+ k_{20}. c_{21}$ | $\frac{dc_{22}}{dt} = k_{20}. c_{21}. k_{21}. c_{22}. c_{23}+ k_{22}. c_{24}+ k_{23}. c_{24}$ |
| $\frac{dc_{7}}{dt} = k_{5}. c_{5}. c_{6}. k_{6}. c_{7}. k_{7}. c_{7}. c_{8}+ k_{8}. c_{9}$ | $\frac{dc_{23}}{dt} = -k_{21}. c_{22}. c_{23}+ k_{22}. c_{24}$ |
| $\frac{dc_{8}}{dt} = -k_{7}. c_{7}. c_{8}+ k_{8}. c_{9}+ k_{11}. c_{11}+ k_{20}. c_{21}$ | $\frac{dc_{24}}{dt} = k_{21}. c_{22}. c_{23}. k_{22}. c_{24}. k_{23}. c_{24}$ |
| $\frac{dc_{9}}{dt} = k_{7}. c_{7}. c_{8}. k_{8}. c_{9}. k_{9}. c_{9}. c_{10}+ k_{10}. c_{11}. k_{15}. c_{9}. c_{17}+ k_{16}. c_{18}$ | $\frac{dc_{25}}{dt} = k_{23}. c_{24}. k_{28}. c_{27}. c_{25}. k_{24}. c_{29}. c_{25}+ k_{25}. c_{30}+ k_{26}. c_{30}$ |
| $\frac{dc_{10}}{dt} = -k_{9}. c_{9}. c_{10}+ k_{10}. c_{11}+ k_{14}. c_{14}$ | $\frac{dc_{26}}{dt} = k_{26}. c_{30}$ |
| $\frac{dc_{11}}{dt} = k_{9}. c_{9}. c_{10}. k_{10}. c_{11}. k_{11}. c_{11}$ | $\frac{dc_{27}}{dt} = p . c_{16}\left( t-\tau\right)- k_{28}. c_{27}. c_{25}$ |
| $\frac{dc_{12}}{dt} = -k_{12}. c_{12}. c_{13}+ k_{13}. c_{14}+ k_{11}. c_{11}$ | $\frac{dc_{28}}{dt} = k_{28}. c_{27}. c_{25}$ |
| $\frac{dc_{13}}{dt} = -k_{12}. c_{12}. c_{13}+ k_{13}. c_{14}+ k_{29}. c_{16}. c_{31}$ | $\frac{dc_{29}}{dt} = -k_{24}. c_{29}. c_{25}+ k_{25}. c_{30}$ |
| $\frac{dc_{14}}{dt} = k_{12}. c_{12}. c_{13}. k_{13}. c_{14}. k_{14}. c_{14}$ | $\frac{dc_{30}}{dt} = k_{24}. c_{29}. c_{25}. k_{25}. c_{30}. k_{26}. c_{30}$ |
| $\frac{dc_{15}}{dt} = k_{14}. c_{14}$ | $\frac{dc_{31}}{dt} = p . c_{16}\left( t-\tau\right)- k_{29}. c_{16}. c_{31}$ |
| $\frac{dc_{16}}{dt} = k_{14}. c_{14}. k_{29}. c_{16}. c_{31}$ |  |

Table 2. TNFR parameters and initial values.^1^

| Name | Species | Initial value (nM) |
| --- | --- | --- |
| $c_{1}$ | TNFα | Model input |
| $c_{2}$ | TNFR1 | 100 |
| $c_{3}$ | TNFα/TNFR1 | 0 |
| $c_{4}$ | TRADD | 150 |
| $c_{5}$ | TNFα/TNFR1/TRADD | 0 |
| $c_{6}$ | TRAF2 | 100 |
| $c_{7}$ | TNFα/TNFR1/TRADD/TRAF2 | 0 |
| $c_{8}$ | RIP-1 | 100 |
| $c_{9}$ | TNFα/ TNFR1/TRADD/TRAF2/RIP-1 | 0 |
| $c_{10}$ | IKK | 100 |
| $c_{11}$ | TNFα/ TNFR1/TRADD/TRAF2/RIP-1/IKK | 0 |
| $c_{12}$ | IKK^*^ | 0 |
| $c_{13}$ | Ik-B/NF-kB | 250 |
| $c_{14}$ | Ik-B/NF-kB/IKK^*^ | 0 |
| $c_{15}$ | Ik-B-P | 0 |
| $c_{16}$ | NF-kB | 0 |
| $c_{17}$ | FADD | 100 |
| $c_{18}$ | TNFα/ TNFR1/TRADD/TRAF2/RIP-1/FADD | 0 |
| $c_{19}$ | TRADD/TRAF2/RIP-1/FADD | 0 |
| $c_{20}$ | Caspase-8 | 80 |
| $c_{21}$ | TRADD/TRAF2/RIP-1/FADD/Caspase-8 | 0 |
| $c_{22}$ | Caspase-8^*^ | 0 |
| $c_{23}$ | Caspase-3 | 200 |
| $c_{24}$ | Caspase-8^*^/Caspase-3 | 0 |
| $c_{25}$ | Caspase-3^*^ | 0 |
| $c_{26}$ | DNA-fragmentation | 0 |
| $c_{27}$ | c-IAP | 0 |
| $c_{28}$ | Caspase-3^*^/c-IAP | 0 |
| $c_{29}$ | DNA | 800 |
| $c_{30}$ | Caspase-3^*^/ DNA | 0 |
| $c_{31}$ | IkB | 0 |

Table 3. TNFR kinetic parameters.^1^

| Kinetic parameter | Value | Kinetic parameter | Value |
| --- | --- | --- | --- |
| $k_{1}$ | 1.85 × 10^2^ | $k_{15}$ | 1.85 × 10^2^ |
| $k_{2}$ | 1.25 | $k_{16}$ | 1.25 |
| $k_{3}$ | 1.85 × 10^2^ | $k_{17}$ | 3.7 × 10^2^ |
| $k_{4}$ | 1.25 | $k_{18}$ | 5 × 10^2^ |
| $k_{5}$ | 1.85 × 10^2^ | $k_{19}$ | 2 × 10^2^ |
| $k_{6}$ | 1.25 | $k_{20}$ | 1 × 10^2^ |
| $k_{7}$ | 1.85 × 10^2^ | $k_{21}$ | 1 × 10^2^ |
| $k_{8}$ | 1.25 | $k_{22}$ | 6 × 10^1^ |
| $k_{9}$ | 1.85 × 10^2^ | $k_{23}$ | 1 × 10^5^ |
| $k_{10}$ | 1.25 | $k_{24}$ | 1.85 × 10^2^ |
| $k_{11}$ | 3.7 × 10^2^ | $k_{25}$ | 1.25 |
| $k_{12}$ | 1.4 × 10^1^ | $k_{26}$ | 3.7 × 10^2^ |
| $k_{13}$ | 1.25 | $k_{27}$ | 3.7 × 10^2^ |
| $k_{14}$ | 3.7 × 10^2^ | $k_{28}$ | 3.7 × 10^2^ |
| $p$ | 1.75 × 10^3^ | $k_{29}$ | 7.5 × 10^5^ |

**EGFR signaling pathway**: Summary of kinetic equations used during development of the multiscale computational model of tumor growth. EGFR signaling was based on the following tables.

Table 4. EGFR equations and parameter values.^2^

| Reaction  number | Rate equation | Parameter values |
| --- | --- | --- |
| 1 | $K_{1}\cdot\left[ R \right]\cdot\left[ TGF\alpha\right]-K_{-1}\cdot\left[ R_{a} \right]$ | $K_{1}= 0.003; K_{-1}= 0.06$ |
| 2 | $K_{2}\cdot\left[ R_{a} \right]\cdot\left[ R_{a} \right]-K_{-2}\cdot\left[ R_{2} \right]$ | $K_{2}= 0.01; K_{-2}= 0.1$ |
| 3 | $K_{3}\cdot\left[ R_{2} \right]-K_{-3}\cdot\left[ RP \right]$ | $K_{3}= 1; K_{-3}= 0.01$ |
| 4 | $V_{4}\cdot\left[ RP \right]/\left( K_{4}+\left[ RP \right] \right)$ | $V_{4}= 450; K_{4}= 50$ |
| 5 | $K_{5}\cdot\left[ RP \right]\cdot\left[ PLC\gamma\right]-K_{-5}\cdot\left[ R\_PL \right]$ | $K_{5}= 0.06; K_{-5}= 0.2$ |
| 6 | $K_{6}\cdot\left[ R\_PL \right]-K_{-6}\cdot\left[ R\_PLP \right]$ | $K_{6}= 1; K_{-6}= 0.05$ |
| 7 | $K_{7}\cdot\left[ R\_PLP \right]-K_{-7}\cdot\left[ RP \right]\cdot\left[ PLC\gamma P \right]$ | $K_{7}= 0.3; K_{-7}= 0.006$ |
| 8 | $V_{8}\cdot\left[ PLC\gamma P \right]/\left( K_{8}+\left[ PLC\gamma P \right] \right)$ | $V_{8}= 1; K_{8}= 100$ |
| 9 | $K_{9}\cdot\left[ RP \right]\cdot\left[ Grb \right]-K_{-9}\cdot\left[ R\_G \right]$ | $K_{9}= 0.003; K_{-9}= 0.05$ |
| 10 | $K_{10}.\left[ R\_G \right]\cdot\left[ SOS \right]-K_{-10}\cdot\left[ R\_G\_S \right]$ | $K_{10}= 0.01; K_{-10}= 0.06$ |
| 11 | $K_{11}\cdot\left[ R\_G\_S \right]-K_{-11}\cdot\left[ RP \right]\cdot\left[ G\_S \right]$ | $K_{11}= 0.03; K_{-11}= 4.5\cdot{10}^{-3}$ |
| 12 | $K_{12}\cdot\left[ G\_S \right]-K_{-12}\cdot\left[ Grb \right]\cdot\left[ SOS \right]$ | $K_{12}= 1.5\cdot{10}^{-3}; K_{-12}= {10}^{-4}$ |
| 13 | $K_{13}\cdot\left[ RP \right]\cdot\left[ Shc \right]-K_{-13}\cdot\left[ R\_Sh \right]$ | $K_{13}= 0.09; K_{-13}= 0.6$ |
| 14 | $K_{14}\cdot\left[ R\_Sh \right]-K_{-14}\cdot\left[ R\_ShP \right]$ | $K_{14}= 6; K_{-14}= 0.06$ |
| 15 | $K_{15}\cdot\left[ R\_ShP \right]-K_{-15}\cdot\left[ ShP \right]\cdot\left[ RP \right]$ | $K_{15}= 0.3; K_{-15}= 9\cdot{10}^{-4}$ |
| 16 | $V_{16}\cdot\left[ ShP \right]/\left( K_{16}+\left[ ShP \right] \right)$ | $V_{16}= 1.7; K_{16}= 340$ |
| 17 | $K_{17}\cdot\left[ R\_ShP \right]\cdot\left[ Grb \right]-K_{-17}\cdot\left[ R\_Sh\_G \right]$ | $K_{17}= 0.003; K_{-17}= 0.1$ |
| 18 | $K_{18}\cdot\left[ R\_Sh\_G \right]-K_{-18}\cdot\left[ RP \right]\cdot\left[ Sh\_G \right]$ | $K_{18}= 0.3; K_{-18}= 9\cdot{10}^{-4}$ |
| 19 | $K_{19}\cdot\left[ R\_Sh\_G \right]\cdot\left[ SOS \right]-K_{-19}\cdot\left[ R\_Sh\_G\_S \right]$ | $K_{19}= 0.01; K_{-19}= 2.14\cdot{10}^{-2}$ |
| 20 | $K_{20}\cdot\left[ R\_Sh\_G\_S \right]-K_{-20}\cdot\left[ Sh\_G\_S \right]\cdot\left[ RP \right]$ | $K_{20}= 0.12; K_{-20}= 2.4\cdot{10}^{-4}$ |
| 21 | $K_{21}\cdot\left[ ShP \right]\cdot\left[ Grb \right]-K_{-21}\cdot\left[ Sh\_G \right]$ | $K_{21}= 0.003; K_{-21}= 0.1$ |
| 22 | $K_{22}\cdot\left[ Sh\_G \right]\cdot\left[ SOS \right]-K_{-22}\cdot\left[ Sh\_G\_S \right]$ | $K_{22}= 0.03; K_{-22}= 0.064$ |
| 23 | $K_{23}\cdot\left[ Sh\_G\_S \right]-K_{-23}\cdot\left[ ShP \right]\cdot\left[ G\_S \right]$ | $K_{23}= 0.1; K_{-23}= 0.021$ |
| 24 | $K_{24}\cdot\left[ R\_ShP \right]\cdot\left[ G\_S \right]-K_{-24}\cdot\left[ R\_Sh\_G\_S \right]$ | $K_{24}=0.009; K_{-24}= 4.29\cdot{10}^{-2}$ |
| 25 | $K_{25}\cdot\left[ PLC\gamma P \right]-K_{-25}\cdot\left[ PLC\gamma P\_I \right]$ | $K_{25}= 1; K_{-25}= 0.03$ |

Table 5. EGFR parameters and initial values.^2^

| Species | Initial value (nM) |
| --- | --- |
| $\left[ TGF\alpha\right]$ | Model input |
| $\left[ R \right]$ | 100 |
| $\left[ R_{a} \right]$ | 0 |
| $\left[ R_{2} \right]$ | 0 |
| $\left[ RP \right]$ | 0 |
| $\left[ R\_PL \right]$ | 0 |
| $\left[ R\_PLP \right]$ | 0 |
| $\left[ R\_G \right]$ | 0 |
| $\left[ R\_G\_S \right]$ | 0 |
| $\left[ R\_Sh \right]$ | 0 |
| $\left[ R\_ShP \right]$ | 0 |
| $\left[ R\_Sh\_G \right]$ | 0 |
| $\left[ R\_Sh\_G\_S \right]$ | 0 |
| $\left[ G\_S \right]$ | 0 |
| $\left[ ShP \right]$ | 0 |
| $\left[ Sh\_G \right]$ | 0 |
| $\left[ Sh\_G\_S \right]$ | 0 |
| $\left[ PLC\gamma\right]$ | 105 |
| $\left[ PLC\gamma P \right]$ | 0 |
| $\left[ PLC\gamma P\_I \right]$ | 0 |
| $\left[ Grb \right]$ | 85 |
| $\left[ Shc \right]$ | 150 |
| $\left[ SOS \right]$ | 34 |

**References**

1. Rangamani, P. & Sirovich, L. Survival and apoptotic pathways initiated by TNF-alpha: Modeling and predictions. *Biotechnol Bioeng* **97**, 1216–1229 (2007).

2. Kholodenko, B. N., Demin, O. V., Moehren, G. & Hoek, J. B. Quantification of short term signaling by the epidermal growth factor receptor. *J Biol Chem* **274**, 30169–30181 (1999).
